# A data-driven framework for identifying patient subgroups on which an AI/machine learning model may underperform

**DOI:** 10.1038/s41746-024-01275-6

**Published:** 2024-11-21

**Authors:** Adarsh Subbaswamy, Berkman Sahiner, Nicholas Petrick, Vinay Pai, Roy Adams, Matthew C. Diamond, Suchi Saria

**Affiliations:** 1 Department of Computer Science, Johns Hopkins University, Baltimore, MD USA; 2 Center for Devices and Radiological Health, U.S. Food and Drug Administration, Silver Spring, MD USA; 3 Department of Psychiatry and Behavioral Science, Johns Hopkins School of Medicine, Baltimore, MD USA; 4 Department of Health Policy and Management, Johns Hopkins Bloomberg School of Public Health, Baltimore, MD USA; 5 Department of Applied Mathematics and Statistics, Johns Hopkins University, Baltimore, MD USA; 6 Bayesian Health, New York, NY USA

**Keywords:** Risk factors, Health policy

## Abstract

A fundamental goal of evaluating the performance of a clinical model is to ensure it performs well across a diverse intended patient population. A primary challenge is that the data used in model development and testing often consist of many overlapping, heterogeneous patient subgroups that may not be explicitly defined or labeled. While a model’s average performance on a dataset may be high, the model can have significantly lower performance for certain subgroups, which may be hard to detect. We describe an algorithmic framework for identifying subgroups with potential performance disparities (AFISP), which produces a set of interpretable phenotypes corresponding to subgroups for which the model’s performance may be relatively lower. This could allow model evaluators, including developers and users, to identify possible failure modes prior to wide-scale deployment. We illustrate the application of AFISP by applying it to a patient deterioration model to detect significant subgroup performance disparities, and show that AFISP is significantly more scalable than existing algorithmic approaches.

## Introduction

Artificial intelligence (AI) and machine learning (ML)-enabled technologies are rapidly entering clinical practice, with more than 800 authorizations of AI/ML-based medical devices by the US Food and Drug Administration (FDA)^1^. AI/ML-based systems have been demonstrated to improve patient outcomes^2,3^ and increase efficiency of healthcare delivery^4^. However, as the adoption of this technology increases, concerns about its bias, robustness, and generalizability have also risen.

A fundamental goal of evaluating the performance of a clinical ML model is to ensure it performs well across a heterogeneous intended patient population. A primary obstacle is that a model may exhibit high overall performance on an evaluation dataset, yet its performance can vary significantly across different subgroups, leading to lower performance for some. This variability can exacerbate disparities in patient care and outcomes, particularly for rare or underrepresented subgroups. Detecting these subgroups can be particularly challenging when they are not explicitly defined (a problem sometimes referred to as hidden stratification^5^).

A variety of case studies have shown how models can learn to predict using dataset-specific artifacts that cause the model’s performance to appear strong, but significantly deteriorate when applied to new sites^6–9^. As one example, the Epic Sepsis Model has been reported to have varying performance when implemented at different hospitals^10,11^. Analysis of factors associated with the model’s performance has shown that it performs worse on patient populations with higher sepsis incidence, comorbidity burden, and cancer prevalence^12^. This information can help determine where models will be safe and effective.

Subgroup performance can also inform post-deployment monitoring, because models can encounter new subgroups not present during initial development and validation^13^. Auditing models for such subgroups is manageable when a small list of relevant subgroups (e.g., different demographic groups) is known beforehand. However, model performance can vary across numerous factors, including patient demographics, comorbidities, and medical history, making it challenging to detect performance differences without labels for many subgroups. Thus, there is a need for tools to help evaluators navigate the possible heterogeneous subgroups where a model may underperform.

Existing methods for identifying subgroups for which a model underperforms typically rely on manual strategies. One approach, schema completion, involves detailed annotation of subgroups^5^. This can be impractical when many complex subgroups exist. Another approach, error auditing, involves manual expert review of misclassified instances, and is also labor-intensive^5,14^. To streamline this process, algorithmic, data-driven approaches have been developed to automatically suggest subgroups for further examination. For tabular data, methods search for subgroups defined by data “slices” (combinations of feature values such as male patients with dementia) with poor performance^15–17^. However, the space of possible slices is vast, often requiring heuristics and parallelization to make searches over slices tractable. Slice-based methods have also been extended to unstructured cross-modal datasets (e.g., paired imaging and text data)^18^.

To address these challenges, we describe AFISP (Algorithmic Framework for Identifying Subgroups with Performance disparities), a scalable, end-to-end approach for identifying subgroups for whom a model may perform poorly. Given a pre-trained model to evaluate, an evaluation dataset, a set of user-specified features (e.g., patient characteristics), and a performance metric, AFISP first identifies the model’s worst-performing subset of the evaluation dataset. This subset reveals shifts in the prevalence of unknown subgroups that would lead to low performance, enabling evaluators to detect mixtures of poorly performing subgroups automatically.

Given the worst-performing data subset, AFISP learns an interpretable characterization (i.e., a concretely defined and easily communicable data slice) of subgroups present within the subset. This provides a way to identify subgroups with potentially poor performance that can be documented and further analyzed. This reduces the reliance on manual review of individual datapoints, and is significantly more scalable than exhaustively searching over all possible subgroups.

In this paper, we illustrate the use of AFISP to algorithmically determine subgroups for which a model has low performance. As a case study, we apply AFISP to a patient deterioration monitoring model and identify several potential subgroups with poor discriminative performance. AFISP finds subgroups similar to the ones found by an exhaustive slice-searching algorithm while being more scalable and running significantly faster. We also demonstrate how identifying problematic subgroups can potentially be used to drive additional data collection and model improvement.

## Results

### Applying AFISP to a patient deterioration monitoring model

**Fig. 1:**
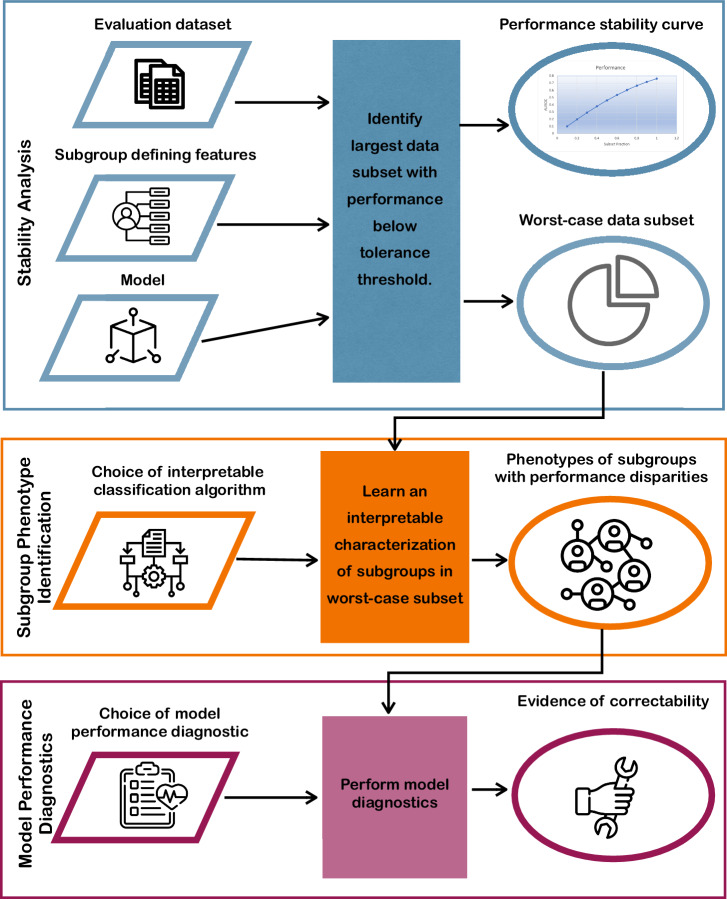
Process diagram depicting the AFISP workflow. AFISP is an end-to-end framework for identifying subgroups on which an ML model may perform poorly. Given a dataset, pre-trained model, and set of user-specified features, the Stability Analysis phase (blue) allows a user to identify the worst-performing subset of the dataset on which the model has significantly deteriorated performance. Then, this data subset is processed to determine concrete subgroup phenotypes (interpretable subgroup descriptions; orange) present within the subset. Finally, an AFISP user applies model performance diagnostics (purple) to each identified subgroup to evaluate if the observed subgroup performance disparity is correctable through changes to the modeling pipeline.

The workflow for applying AFISP is outlined in the process diagram in Fig. 1. AFISP takes as input a model to evaluate, an evaluation dataset, and a set of possible subgroup-defining features.

In our experiments, we used AFISP to evaluate a model inspired by the advanced alert monitor (AAM)^19^, an ML model trained to detect inpatient deterioration outside of the intensive care unit (ICU) that was shown to decrease patient mortality when deployed at 19 hospitals^3^. Note that we have implemented an AAM-inspired model for illustrative purposes, so our findings do not represent subgroups on which the original AAM model underperforms. AFISP uses an evaluation dataset, for which we used 60,998 patient encounters extracted from the electronic health records of adult patients admitted to any of 5 hospitals within the Johns Hopkins Hospital network (excluding labor and delivery admissions) from 2016 to 2021.

The final input to AFISP is a set of user-chosen features that are used to create subgroup definitions. Some of these features may be inputs used by the model to make predictions, while others may represent different characteristics that the user wants to evaluate for potential disparities. Prior studies demonstrating clinical predictive model performance disparities between subgroups have primarily focused on demographic subgroups, such as performance disparities by race or sex^20,21^. By contrast, in the current study we examine the potential existence of more broadly defined patient subgroups for which the AAM-inspired model underperforms. We allow for the discovery of subgroup *phenotypes* (i.e., the observed characteristics of a subgroup) defined with respect to features pertaining to over 80 comorbidities (e.g., prostate cancer, chronic bronchitis, etc.), patient demographics (age and sex), hospital information (trauma level and hospital size), and admission circumstances (including admission source and admission time and season). In total, 91 features were selected.

We measured performance in terms of the AAM-inspired model’s ability to discriminate between patients who deteriorated outside of the ICU vs. patients who did not deteriorate. Specifically, as defined in the AAM development paper^19^, the outcome was positive if a patient had an in-hospital death, an unexpected transfer from the ICU (i.e., less than 6 h ICU stay followed by transfer to the operating room), or a long stay in the ICU (i.e., greater than 6 h ICU stay not due to scheduled surgery). We selected the area under the ROC curve (AUROC) as an overall measure of discriminative performance.

### Analyzing the stability of model performance as subgroup prevalence changes

**Fig. 2:**
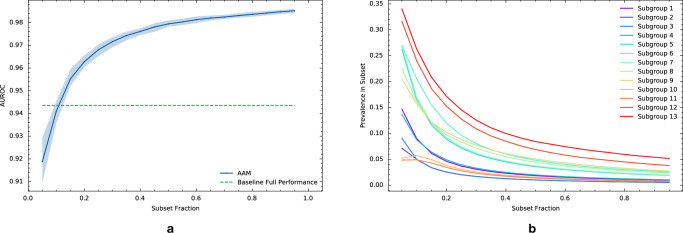
Performance analysis of the AAM-inspired model as subgroup prevalence changes. **a** Performance stability curve for the AAM-inspired model under a shift in patient subgroup prevalence as measured by area under the ROC curve (AUROC). The blue curve plots the AUROC of the AAM-inspired model on the worst-case subset of a given size (x-axis; the fraction of the whole evaluation dataset). The worst-case subset is determined algorithmically based on the AAM-inspired model’s expected loss conditional on a set of 91 patient characteristics. The shaded region denotes a 95% bootstrap confidence interval derived from 100 resamples. The green dashed line denotes a minimum performance threshold that was selected based on the performance of an existing model. **b** Prevalence of subgroups with poor performance extracted by AFISP vs. subset fraction. While these subgroups are rare in the full dataset (subset fraction = 1), they become more common in smaller worst-case subsets.

We analyzed how the performance of the AAM-inspired model decays as the evaluation data distribution is gradually changed adversarially through shifts in the prevalence of subgroups defined with respect to the 91 features described above. The results are depicted in the performance stability curve in Fig. 2a. The blue curve shows how the performance (AUROC) of the AAM-inspired model on the worst-performing subset of the evaluation dataset varies vs. the size (as a fraction of the number of evaluation dataset samples) of the worst-performing subset. For a given subset fraction *α*, a corresponding worst-performing data subset is identified by the algorithm such that it contains the 100 × *α*% samples in the evaluation dataset with the worst expected loss (i.e., worst average loss conditioned on the selected 91 features). A subset fraction of *α* = 1 corresponds to model performance on the full evaluation dataset, and smaller data subsets (i.e., smaller *α*) can statistically differ more from the full population than larger subsets.

As expected, the AUROC of the AAM-inspired model decreases as the subset fraction gets smaller, since smaller worst-performing data subsets correspond to worse quantiles of the AUROC. On the full evaluation dataset (i.e., subset fraction of 1), the AAM-inspired model achieves an AUROC of 0.986 (CI 0.985, 0.987). As the subset fraction becomes smaller, the AAM-inspired model’s AUROC decays to 0.919 (CI 0.910, 0.929) at a fraction of 0.05.

To select a specific subset to analyze, we used a user-determined performance threshold. This threshold can be determined in a variety of ways, including from published literature, standard of care, or based on existing models. For illustration, we trained a “baseline” model to serve the role of an existing model (details in Supplementary Information 1C), and used its performance on the evaluation set as the minimum performance threshold for subgroup performance in the AAM-inspired model. Using this threshold (AUROC of 0.944; the green horizontal line in Fig. 2), we found that subset fractions of size ≈10% or less yielded a worse AUROC.

### Learning interpretable subgroup phenotypes that produce poor model performance

Given the worst-performing data subset, we want to determine specific subgroups present within the subset on which the AAM-inspired model has performance worse than the reference value. We used SIRUS^22^, a rule-based classification algorithm, to determine interpretable subgroup phenotypes (i.e., combinations of values of the 91 selected features). We allowed for up to three features to be simultaneously considered in a phenotype definition. After filtering subgroups based on significance (and correcting for multiple comparisons) and effect size, AFISP recovered the 13 subgroups reported in Table 1 (ordered by within-subgroup AUROC). The subgroups are all quite rare in the evaluation dataset, with the most prevalent subgroup (Subgroup 13) making up only 4.4% of the evaluation dataset. We also plot the prevalence of these subgroups in the worst-performing subsets extracted during the stability analysis stage in Fig. 2b. This shows that worse model performance is associated with the increasing prevalence of these subgroups.

**Table 1:** Poorly performing subgroups identified by AFISP

Subgroup #	Phenotype	AUROC [95% Bootstrap CI]	Number of patients
AFISP 1	Anemia and nonspecific lung disease	0.81 [0.78, 0.84]	562
AFISP 2	Nonspecific lung disease and Hypoxemia	0.82 [0.77, 0.86]	310
AFISP 3	Sepsis and acute respiratory failure	0.83 [0.79, 0.87]	306
AFISP 4	Sepsis and anemia	0.85 [0.82, 0.88]	606
AFISP 5	Acute respiratory failure and no bronchitis	0.89 [0.88, 0.91]	1095
AFISP 6	Nonspecific lung disease and hypoxemia	0.89 [0.88, 0.91]	1468
AFISP 7	Acute respiratory failure	0.89 [0.88, 0.91]	1137
AFISP 8	Sepsis	0.91 [0.90, 0.93]	1361
AFISP 9	Hypoxemia	0.91 [0.90, 0.93]	1587
AFISP 10	Nonspecific lung disease and not admitted from ED	0.92 [0.90, 0.94]	468
AFISP 11	Nonspecific lung disease and no infection	0.92 [0.90, 0.95]	422
AFISP 12	Transferred from nursing facility and admitted from ED	0.93 [0.91, 0.94]	2196
AFISP 13	Transferred from a nursing facility	0.93 [0.92, 0.94]	3035

Phenotypes of subgroups found by AFISP in the worst 10% subset of the evaluation dataset, and the performance of the AAM-inspired model on these subgroups. For reference, the full evaluation dataset contained 60,998 patients and the AUROC of the AAM-inspired model on the full dataset was 0.986 [0.985, 0.987]. Confidence intervals computing using 100 bootstrap resamples. All subgroups had a statistically significant difference (at the 0.05 significance level) in performance from the performance threshold (0.944) with all *p* values less than 1 × 10^−4^ after correcting for multiple comparisons.

Notably, four of the subgroups have AUROC intervals that are below 0.9, and there is some redundancy in the specified feature values. Further, nine of the subgroups have multivariate phenotype definitions. Thus, any univariate subgroup analysis would fail to identify these subgroups. We also note that except for choosing the 91 features for possible subgroup definitions, the process of producing Table 1 was automated and did not involve a human user’s oversight.

### AFISP identifies subgroups similar to those found by exhaustive algorithmic approaches

Without ground truth labels for subgroups with poor performance, it is difficult to assess the quality or accuracy of the subgroups found by AFISP. Thus, we instead compared the subgroups found by AFISP to those found by Slice Finder (SF)^15^, a state-of-the-art algorithmic approach which searches all possible degree two slices (i.e., subgroup phenotypes involving at most two features). As expected of an exhaustive search of 34,716 possible slices, SF found 748 slices corresponding to subgroups with poor performance—substantially more than AFISP identified. However, these 748 slices were a superset of the 13 found by AFISP.

To visualize and compare the similarity of subgroups found by AFISP and SF, we used partial least squares (PLS) regression^23^ to jointly project the AFISP subgroups, SF slices, and a random sample of 2000 of the possible 34,716 slices into a two-dimensional vector space that captures correlations between subgroups and performance within those subgroups.

**Fig. 3:**
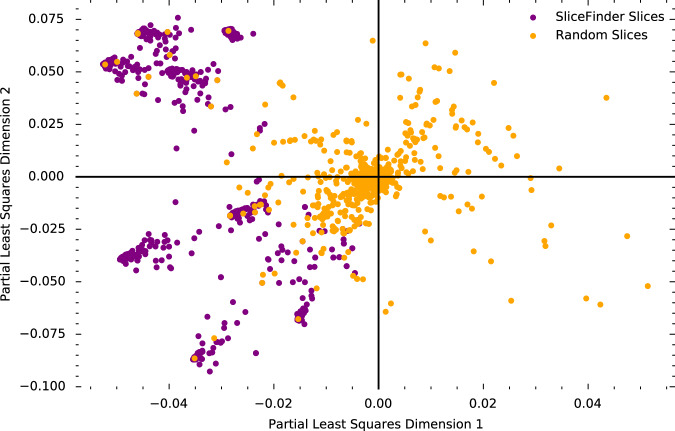
Vector embeddings of patient subgroups. Loading plot of the first two dimensions created by jointly embedding subgroups found by SliceFinder (purple points), random candidate subgroups (orange points), and subgroups found by AFISP (not pictured) using partial least squares (PLS) to predict model loss. While the random slices are spread throughout the space, all SliceFinder slices are in the left two quadrants, indicating that PLS was able to capture subgroup-performance correlations.

To see that the vector space captures subgroup performance correlations, we plot the PLS representations of the SF slices and 2000 randomly selected slices from the set of all possible slices in Fig. 3. The SF slices, which correspond to those found by SF to have poor performance, are all in the left two quadrants, while the randomly selected slices are distributed across the space. Thus, the left region of the space captures slices with poor performance.

**Fig. 4:**
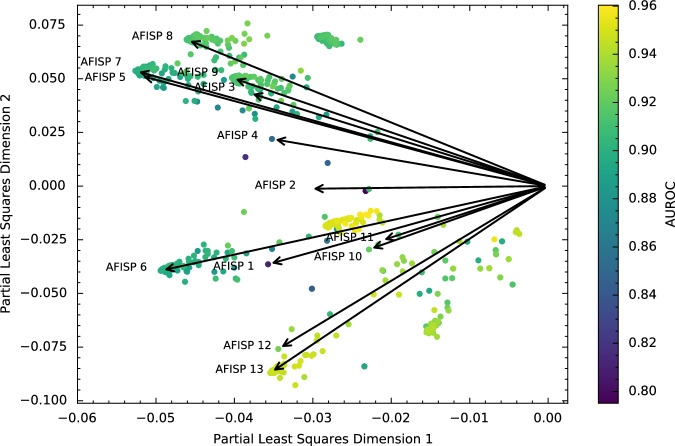
Comparing vector embeddings of subgroups found by AFISP and SliceFinder. Loading plot of the same partial least squares (PLS) regression as in Fig. 3, this time plotting the subgroups found by SliceFinder as points and the subgroups found by AFISP as vectors (black arrows). We plot the AFISP subgroups as vectors to enable a better visual assessment of cosine similarity: a smaller angle between subgroups indicates more similarity. Thus, points close to or along a vector are SliceFinder subgroups that are highly cosine similar to the corresponding AFISP subgroup. The points are colored according to the AAM-inspired model’s AUROC within the corresponding subgroup.

Next, in Fig. 4, we plot the SF slices as points (colored according to AUROC) and the AFISP subgroups as vector directions in the space (black arrows). This allows (cosine) similarity between subgroups to be assessed using the angle between two points/vectors (with a smaller angle indicating more similarity). We see that the plot captures similarity amongst rules. For example, the vectors for AFISP subgroups 3, 5, and 7 are closely aligned, and all of these subgroups correspond to subsets of patients with acute respiratory failure. Overall, most of the 748 Slice Finder subgroups are similar to at least one of the AFISP subgroups. A few patches of Slice Finder subgroups are outliers, but the corresponding AUROCs of these rules are relatively higher (≈0.9 or more). Thus, AFISP found a concise set of subgroups that covers most of the slices selected by SF.

To further validate the proposed approach, in the Supplementary Information we compare subgroups found by AFISP to those found by SF on an implementation of the SICULA ICU mortality prediction model^24^ using the MIMIC-III dataset^25^.

### Assessing the correctability of subgroup performance disparities

**Fig. 5:**
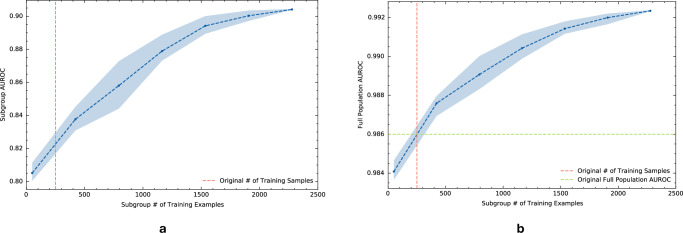
Testing model performance after retraining with more data. **a** Subgroup performance learning curve for subgroup 2 found by AFISP: `Patient has anemia & patient has a nonspecific lung disease'. The learning curve plots the subgroup AUROC of the model vs. the amount of training data present from the subgroup. The amount of non-subgroup data is kept fixed. **b** Full population performance learning curve for this subgroup. This plots the full population AUROC of the model vs. the amount of training data present from the subgroup. In both plots, the points represent the average of ten samples of subgroup data, and the shaded region denotes a 95% confidence interval. The vertical dashed line denotes the amount of training samples used for the original model. The green horizontal dashed line is the full population AUROC of the original model.

An important component in determining the safety risk associated with poor subgroup performance is to use model performance diagnostics to assess the correctability of an observed performance gap. As just one example, considering the worst-performing subgroup in Table 1, we tested the effect of adding more training data from the subgroup while keeping the amount of training data from outside the subgroup fixed. Figure 5a shows the effect of additional training data from the subgroup on the within-subgroup AUROC. The vertical dashed lines denote the amount of subgroup data present in the original training set used to develop the model. Subgroup data beyond that amount was taken from the training datasets for the other hospitals. The plot demonstrates that, in this case, data collection would be helpful in addressing the performance gap. With 2000 training samples from the subgroup, the performance would be comparable to that of many of the other subgroups in Table 1. Figure 5b shows that increasing the amount of training data for just this single subgroup also improved the model’s AUROC on the full evaluation dataset.

### Validating AFISP accuracy and scalability

The application of the AAM-inspired model illustrates the usability of the AFISP workflow and qualitatively shows that AFISP can concisely capture important subgroups found by exhaustive search-based approaches. To validate the correctness of the subgroup discovery method we turned to synthetic data in which we created problematic subgroups and measured the ability of methods to recover these subgroups. Specifically, we simulated a classification task with ten binary features, and trained a well-specified logistic regression prediction model. We then created a problematic subgroup for this model by randomly flipping labels in samples corresponding to a subgroup defined by two features.

**Fig. 6:**
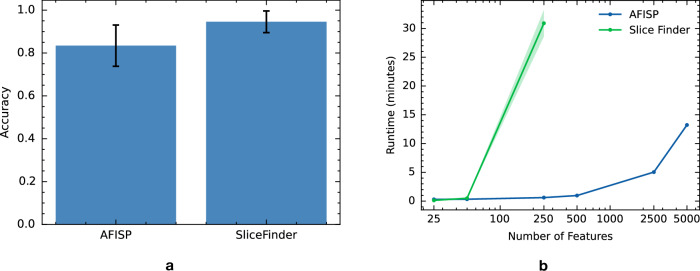
Accuracy and efficiency of subgroup discovery methods on simulated data. **a** Results from a synthetic data experiment in which a poorly performing subgroup was manually defined for the given prediction model. The bar plot reports AFISP and SliceFinder’s accuracy (percent of times that each method found the problematic subgroup). Error bars represent 95% Wilson score intervals from 50 trials. **b** Runtime in minutes of each method vs the number of binary features (on log scale). Shaded regions represent one standard error from five repetitions.

Applying AFISP and SliceFinder to a holdout evaluation set of 10,000 samples (and repeating 50 times), we report the accuracy (percent of times that each method found the problematic subgroup) of each method in Fig. 6a. Due to the exhaustive nature of its search, SF found the problematic subgroup 94.6% (CI 89.5, 99.6) of the time. By contrast, AFISP found the problematic subgroup 83.4% (CI: 73.8, 93.0) of the time (equality of two proportions test *p* value of 0.073).

While SF was more accurate in subgroup discovery than AFISP, we also evaluated the scalability of each method as the number of features increased from 25 to 5000. In Fig. 6b, we plot the runtime (in minutes) of each method vs the number of features, as measured on a server with 64 CPU cores. As expected, the runtime of SF scales much worse than that of AFISP. SF’s scaling would be even worse if SF considered slices involving more than two features, and if the features had more than two categories. Thus, for datasets with large numbers of features, high cardinality features, or slices of high degree, exhaustive search-based methods like SF quickly become intractable.

While the output of AFISP is similar in form to the output of existing search-based algorithmic approaches like SliceFinder, there are a few key differences. First, while an exhaustive search is more thorough (and thus more likely to find all poorly performing subgroups), it may find too many subgroups (e.g., have issues with redundancy) and it scales poorly. For the purposes of hypothesis generation and exploratory data analysis, an approach like AFISP may be more suitable for quickly identifying significantly underperforming subgroups. Second, the intermediate outputs of AFISP have additional value beyond what is possible with approaches like SliceFinder. In particular, the performance stability curve in Fig. 2a serves as a detector that tests for the existence of some subgroups with poor performance. For example, if this curve were to be near constant, this would indicate that there is no detectable underperforming subgroup with respect to the given user-selected features. This is noteworthy because in cases where we are not able to identify specific phenotypes or slices (e.g., because the phenotype template is a poor fit for the true underlying subgroups), AFISP’s performance stability curve may still be able to detect the existence of the subgroups.

## Discussion

In healthcare, evaluating a predictive ML model to ensure its safety and effectiveness across the various populations and subgroups to which it will be applied is of primary importance. Ideally, this is done during the initial evaluation of the model and prior to model deployment to avoid poor and possibly unsafe performance for any group. However, this is currently a major challenge due to the complex nature of the prediction algorithms, the existence of hidden stratification and a priori unknown subgroups, and the burden of expert-driven manual error review^5,26^. In this study, we described and demonstrated the potential of AFISP, a scalable algorithmic, data-driven approach for identifying subgroups for which a model may underperform. Applying this data-driven approach to our custom implementation of AAM, an inpatient deterioration prediction system, we identified 13 subgroups with significant gaps in discriminative performance (e.g., AUROC of 0.81 in subgroup 1 vs. 0.986 on average) with minimal user input. We also demonstrated that we could use model performance diagnostics to assess the correctability of a subgroup performance gap found by AFISP. We found the performance gap in the worst-performing subgroup for the AAM-inspired model to be largely fixable through a targeted collection of more data from that subgroup.

We also showed across real data examples (see Supplementary Information Section 2 for an additional real data example) and in a simulation study that subgroups found by AFISP were similar to those found by exhaustive search-based methods like SliceFinder (SF). The results present an interesting trade-off: SF can more accurately find all underperforming subgroups, but scales very poorly in the number (and cardinality) of candidate factors. By contrast, AFISP scales better but only finds a representative subset of underperforming subgroups. The improved scalability is advantageous because model performance can vary with respect to numerous factors, including demographic information, but also patient history, comorbidities, circumstances of admission, geographic location, hospital type, data collection instruments, etc. By having the ability to include many possibly relevant factors into the feature set, model evaluators can avoid having to define all relevant subgroups to test ahead of time.

On the other hand, from a safety perspective, evaluators would want to identify all underperforming subgroups. However, as our experiments show, there can be significant redundancy between underperforming subgroups because of overlapping features. For example, eight out of 13 AFISP subgroups involve either a nonspecific lung disease or acute respiratory failure (two conditions which share the same ICD-9 heading). This redundancy is even worse for the 748 subgroups identified by SliceFinder, for which many correspond to highly overlapping groups of patients. For exploratory purposes and hypothesis generation, this redundancy could result in a large overhead for the expert who needs to review the identified subgroups. A useful avenue for future work would be to learn subgroups that can leverage hierarchical structure (such as relationships between ICD-9 codes) to reduce the amount of repetition or to provide subgroup definitions at varying levels of granularity.

Algorithmic evaluation methods such as AFISP could provide significant value to how model evaluations are performed throughout a model’s lifecycle. For example, during model validation, prior to deployment, model developers can perform algorithmic subgroup discovery to proactively identify possible failure modes for the model. Additionally, AFISP could help with monitoring a model’s performance post-deployment. As new model performance data becomes available from active sites, algorithmic subgroup discovery can be run in order to identify new vulnerable subgroups that were not previously present or were too small to be reliably detected. This complements real-time monitoring procedures that actively audit individual predictions for errors as they occur during use^27,28^.

With regard to model improvement and updating, we demonstrated a model performance diagnostic that measured the ability of additional subgroup-specific data collection and model retraining to correct subgroup performance disparities. However, in some cases this model diagnostic may be challenging to perform because of difficulties in collecting targeted data. Another reason for performance disparities can be due to model underfitting^29^. Model developers can attempt to diagnose this by seeing if changes to the type of model used (e.g., using tree-based ensembles instead of linear models) or to the richness of the feature set (e.g., adding new features or including interactions) improve the disparity. Methods for updating models used by humans^30,31^ and model localization through transfer learning^32,33^ remain open areas of research.

There are some limitations: First, the proposed methodology is limited by the heterogeneity of the available evaluation data. AFISP only uncovers latent subgroups present in the data. Thus, if there is a subgroup with poor model performance that is not represented in the evaluation data, the method will fail to identify this subgroup. For example, if a model has very poor performance on patients with HIV/AIDS, but the evaluation dataset contains no (or few) patients with HIV/AIDS, then the algorithm would be unable to discover the model’s under-performance on the HIV/AIDS patient subgroup. This limitation underscores the importance of collecting diverse, high-quality datasets for performing an algorithmic evaluation. Additionally, the study data was limited to hospitals within the same institutional network. Thus, subgroups identified by AFISP could not be validated on out-of-network data.

Second, distinguishing the signal (i.e., subgroups with poor performance) from noise (i.e., small arbitrary collections of samples with high variance in performance) is difficult when the evaluation dataset is small, or the subgroup in question is very rare in the evaluation dataset. In such cases, the confidence interval for a subgroup performance estimate will be large, indicating high uncertainty for small-sized subgroups. Fortunately, in our experiments, the evaluation dataset contained more than 60,000 samples, and the smallest subgroup phenotype that was identified contained more than 300 patients. Additionally, the confidence intervals plotted in the stability curve (Fig. 2) and computed for the phenotypes (Table 1) are sufficiently narrow such that differences from the reference performance threshold are statistically significant. More generally, the variance of a performance estimate can provide additional information about the potential relevance of the subgroup.

Finally, it is important that a holistic model evaluation account for how a model is intended to be used. In our case study, we trained and evaluated the AAM-inspired model using only an encounter-level analysis. In practice, systems for inpatient deterioration monitoring make real-time predictions over the course of a patient’s stay. Thus, in addition to a model’s encounter-level ability to discriminate between patients who deteriorate and patients who do not, it is important to evaluate the alert-level performance of the model to identify subgroups where the model fails to alert (i.e., low recall) and subgroups where the model over-alerts and creates process delays (i.e., low precision). Repeating the proposed analysis with other relevant performance metrics and shift types can provide a more comprehensive assessment of possible failure modes.

Despite these limitations, we posit that the current study represents a significant advance in the ability of model developers and evaluators to better assess the safety and effectiveness of a model across a range of populations. The AFISP framework shows that the available evaluation data (a resource that is often limited) can be used to provide a more detailed breakdown of model performance, to understand model performance as the underlying data distribution changes, and to identify subgroups on which the model may underperform. Nevertheless, we emphasize that a data-driven framework like AFISP cannot be used in a vacuum. It provides tools meant to assist human experts as they perform a thorough evaluation of model performance. Experimentation with this and related frameworks to a larger set of AI models has the potential to refine the way AI models are developed and evaluated across a broad set of applications. In this way, frameworks such as AFISP can aid human teams in ensuring that safe and effective devices of public health importance are made available for a diverse patient population.

## Methods

### Stability analysis

Hidden stratification is difficult to detect because it is characterized by a disparity between a model’s average performance and its performance on sufficiently rare, but a priori unknown, subgroups. Stability analysis is a powerful tool for surfacing these types of subgroups because it allows one to test the uniformity of a model’s performance across a range of different data distributions^34^. Thus, by defining a set of data distributions that vary by subgroup prevalence, a model evaluator can use stability analysis to determine if there exist data distributions within the set on which the model has problematically low performance compared to its average performance on the full evaluation data distribution.

The first step of the AFISP framework is to perform a stability analysis to identify the largest worst-performing subset of the evaluation data on which the model’s performance is below a user-defined threshold. This subset is then further analyzed to determine concrete subgroups that are present within the identified data subset.

#### Identifying worst-case subsets

We use a stability analysis framework developed by ref. ^34^ to perform the first step of AFISP. We will refer to this method as SA (stability analysis).

In the first step, our goal is to identify the subset of the full evaluation dataset of a particular size, and defined by a particular set of user-selected features, on which the model performs worst. For example, if a user is interested in evaluating performance across demographic subgroups, they might allow the subgroup definition to depend on demographic characteristics such as age, sex, and race. Formally, for an evaluation dataset *D* consisting of input features *X* and prediction label *Y*, the user specifies a set of features *W* ⊂ {*X*, *Y*} and a subset fraction *α* (subset size measured as a fraction of the dataset). Then, following SA^34^, we define an *uncertainty set* made up of all possible subsets of size *α* defined based solely on features in *W*.

##### Definition 1

(Uncertainty set). Let ${\mathcal{W}}$ be the sample space of *W* and let *P* be the distribution of the evaluation data. Then, define the **uncertainty set** as ${{\mathcal{U}}}_{\alpha }=\{{\mathcal{S}}\subseteq {\mathcal{W}}:P(W\in S)=\alpha \}$ or the collection of subsets of values and features in ${\mathcal{W}}$ with probability *α* under *P*. Note that ${\mathcal{S}}$ denotes subsets of the sample space of *W*, and $S\in {\mathcal{S}}$ represents an individual such subset.

Considering demographic subgroups again, a user might select *W* = age, race, sex, and *α* = 20%. The corresponding uncertainty set ${{\mathcal{U}}}_{\alpha }$ would contain subsets of the demographics space such that 20% of samples are included in each subset. Across the subsets, the way other variables relate to variables in *W* does not change. For example, considering a subset of Black men under 30, the comorbidity distribution in this subgroup would be unchanged. However, if we also included comorbidities in *W*, the uncertainty set could hypothetically contain a subset that includes only Black men under 30 who have either sickle cell disease or type 1 diabetes. Note that when using SA, one cannot select subsets that are not represented in the evaluation distribution. For example, if the evaluation dataset contains no male patients over age 70, then neither will subsets in ${{\mathcal{U}}}_{\alpha }$. While ideally we would like to consider subgroups not represented in the evaluation dataset, SA is statistically constrained to only uncovering unknown subgroups that are present within the evaluation dataset.

#### Finding the worst-performing subset in ${{\mathcal{U}}}_{\alpha }$

Once the uncertainty set has been defined, SA finds the subset in ${{\mathcal{U}}}_{\alpha }$ that has the *worst* average performance under a model ${\mathcal{M}}$ and for a particular loss function *ℓ*. Formally, this is defined by the following optimization problem:

1 $$\mathop{\sup }\limits_{S\in {{\mathcal{U}}}_{\alpha }}{{\mathbb{E}}}_{P}[\ell ({\mathcal{M}}(X),Y)| W\in S].$$

In words, SA tries to find *S* in ${{\mathcal{U}}}_{\alpha }$ that maximizes the expected loss on the distribution of the evaluation data subset *S*. This is referred to as the *worst-performing subset* of size *α*. For details regarding how this optimization problem is solved, we refer readers to the SA paper^34^.

The worst-performing subset will likely be a mixture of different subgroups, with the prevalence of each of the constituent subgroups different from its prevalence in the evaluation dataset. As an example, suppose Black females under 18 make up 8% of patients in the evaluation dataset. It is possible for the worst-performing subset of size 0.1 to consist of 60% Black females under 18. By examining the makeup of worst-performing subsets as a function of size, we can determine subgroup characteristics that are associated with poor model performance.

#### Identifying a data subset with poor performance

Solving the optimization problem in Equation (1) provides evaluators a way to study how model performance decays as the evaluation population distribution is gradually changed adversarially (with respect to the target model). Given a set of shift characteristics *W*, a subset size of *α* = 1 corresponds to model performance on the full evaluation dataset. As *α* decreases and approaches 0, the worst-performing shifted data distribution is allowed to be more and more different from the original data distribution (smaller subsets can differ more from the overall population than larger subsets). Thus, for a fixed choice of *W*, we can plot a performance stability curve of the worst-case shift performance for a grid of values $\alpha \in \left(0,1\right]$.

Applying SA for a grid of *α* values, we created the stability curve presented in the Results section in Fig. 2. The performance of the target model (in blue) decays as the subset fraction decreases. Using the performance threshold defined by the baseline model’s performance, we identified the largest subset fraction that produces performance worse than the threshold to be *α* = 0.1. The performance threshold can be determined in several ways, including using a known tolerance (i.e., the model is not suitable if its performance is below a certain value) or using reference values from a widely used or previously approved baseline model. The worst-performing data subset selected through this procedure contains subgroups that occur at a higher prevalence than in the full dataset. In cases where a reference performance threshold cannot be established, one could instead select the subset size producing the largest *effect size* (e.g., the difference between loss in and out of the worst-case subset normalized by the pooled standard deviation of the loss).

### Subgroup phenotype learning: interpretably characterizing subgroups

Once the worst-performing subset has been determined, we are interested in understanding the specific characteristics of the subset that are associated with poor model performance. With respect to the user-specified subgroup features *W*, this means finding combinations of values of *W* that result in poor model performance. We will refer to such a specification of *W* values as subgroup phenotypes—the observed characteristics of a subgroup.

As part of this approach, it is important that phenotypes satisfy three primary criteria: interpretability, accuracy, and generalizability. First, when phenotypes are interpretable, they can be easily documented and communicated to model developers, users, and overseers. Second, when phenotypes are accurate, they precisely describe individuals found in the subset identified in the first step. Third, when phenotypes are generalizable, they are not overfit to a particular dataset. The goal of this step of the framework is to learn subgroup phenotypes from the evaluation data that satisfy these desired criteria. In practice, we formulate this as a classification task, in which we use interpretable ML classifiers to distinguish between samples in the evaluation dataset that are in the worst-case subset and samples that are not, using the feature set *W*.

Many methods for interpretable classification exist and could potentially be used within the AFISP framework^35^. We chose SIRUS^22^ because it produces phenotypes that satisfy our three criteria. SIRUS is interpretable by design because the algorithm consists of a *decision rule set*. A decision rule is a classifier corresponding to an if-then-else statement that reads “if *condition* then *response* else *default response*”. The *condition* is a logical conjunction (i.e., “and” statement) that forms a phenotype. For example, if *W* = {age, race, sex}, a possible *condition* would be “age >75 AND *race* is White AND sex is female.” A decision rule set is an ensemble of decision rules. Thus, we extract phenotypes from a classifier fit using SIRUS by taking the *condition* from each rule in the ensemble. To determine which of the phenotypes were associated with poor performance, we performed one-sided *z*-tests^36^ to test if 100 bootstrap resampled AUROCs within the subgroup phenotype were significantly lower than the performance tolerance threshold chosen during the Stability Analysis stage. To correct for multiple testing, we applied the Holm–Bonferroni method, setting the family-wise error rate to 0.05. Following the practices of existing algorithmic subgroup discovery approaches^15^, and due to the large subgroup sizes leading to small *p* values, we also filtered rules by effect size. Specifically, we computed Cohen’s $d=\frac{{\bar{x}}_{1}-{\bar{x}}_{2}}{s}$ (where $\bar{x}$ is the mean expected conditional loss in a subgroup and *s* is the pooled standard deviation) and took rules with *d* ≥ 0.4.

We found that SIRUS accurately captured members of the worst-case subset in our experiments. Computing metrics using fivefold CV, SIRUS achieved a mean AUROC of 0.895 (SD 0.008) on the worst-case subset membership task. Picking thresholds for each fold to fix sensitivity (i.e., recall) at 0.8, SIRUS yielded a mean specificity of 0.863 (SD 0.026). We note that decision rule sets are a highly flexible model class, and that the rules used by SIRUS are extracted from decision paths in a Random Forest (i.e., SIRUS can achieve similar accuracies to Random Forest models, while being much more interpretable).

The final quality of phenotypes found by SIRUS is their generalizability. Multiple, distinct rule sets could be used to accurately classify the members of the worst-case subset. Thus, there is the potential for *instability* in that the algorithm could return different rule sets across different runs of the algorithm or across different samples from the same distribution. This would harm the reproducibility of analyses done using the learned phenotypes. Fortunately, SIRUS contains a hyperparameter, ${p}_{0}\in \left(0,1\right]$, which is a rule extraction threshold that defines the proportion of trees in a Random Forest that a rule needs to appear in to be allowed in the rule set. This thresholding mechanism can make rules found by SIRUS more generalizable because they appear in multiple, independently constructed decision trees. We used the cross-validation procedure suggested by the SIRUS authors to set *p*_0_ = 0.022.

### Model performance diagnostics

The output of the previous steps is sufficient to create initial documentation of potential safety and effectiveness concerns related to the model’s performance on the identified subgroups. Model performance diagnostics are a suite of techniques that model developers can use to determine the correctability of poor model performance in a subgroup. The motivation is that if a model developer can correct an observed subgroup performance disparity, then that subgroup is no longer a concern. If not, then further effort would be important to determine if the subgroup is of high clinical relevance and if the performance disparity represents a substantial practical risk. While a variety of diagnostics exist^29^, we elected to use a particularly simple diagnostic to demonstrate the AFISP framework.

We chose to examine the potential for targeted data collection to improve model performance on a subgroup. To do this, we plot a *learning curve* of the model’s performance on the subgroup vs. the number of training examples from that subgroup. This is done by keeping the non-subgroup training data fixed and adding to it randomly subsampled training data of a specific size from the subgroup. The model is then retrained on the dataset, and the subgroup-specific performance (across multiple random subsamples) is averaged and plotted. The learning curves produced from our experiments are shown in Fig. 5. The additional subgroup training data for our diagnostic was taken from training datasets for hospitals that were not used to train the AAM-inspired model in the primary experiments. For this aspect of the framework to be most useful, it is important for more data to be collectible and available from the identified subgroups. More information is in the Experimental details section.

### Comparing subgroup similarity

To compare the similarity of the subgroups found by AFISP and Slice Finder, we jointly embedded the AFISP and Slice Finder subgroups into a low-dimensional vector space. We wanted the representation to preserve two types of relationships about subgroups: We wanted to capture overlap in the individuals covered by different subgroup phenotypes, and we wanted to capture correlations between subgroups and the AAM-inspired model’s performance within those subgroups. Given these criteria, we used Partial Least Squares regression (PLS) (as implemented in the scikit-learn package^37^) to embed the subgroups. PLS is a method for linear regression of a dependent variable *Y* on the principal components of an input matrix *X*.

To apply PLS, we first defined a subgroup indicator matrix $X\in {\{0,1\}}^{N\times \left(m+k+r\right)}$ such that each row $i\in \left[1,N\approx 68000\right]$ corresponds to an individual in the evaluation dataset, and each column $j\in \left[1,m+k+r\right]$ is a binary indicator for whether individual *i* is present in subgroup *j* for each of the *m* = 13 AFISP subgroups, *k* = 748 Slice Finder subgroups, and *r* = 2000 subgroups sampled from the 34,716 possible slices. We defined *Y* to be the *N* × 1 vector of expected conditional losses for each individual. Fitting a PLS regression with 2 components to *X* and *Y* yields a *loading matrix* $L\in {{\mathcal{R}}}^{\left(m+k+r\right)\times 2}$ which maps each of the subgroups in the *X* matrix to a two-dimensional vector representation. Given vector representations for two subgroups, their similarity can be quantified through their cosine similarity (cosine of the angle between the vectors). Two vectors are maximally similar if their directions are aligned (i.e., cosine similarity is 1), while two vectors are maximally dissimilar if they are orthogonal (i.e., cosine similarity is 0). The *loading plot* of this matrix is shown in Figs. 3, 4.

### Experimental details

#### Real data

This study was approved by the Johns Hopkins University internal review board (IRB no. 00278092), and a waiver of consent was obtained. We trained and evaluated our custom implementation of an AAM-inspired model using anonymized data extracted from the electronic health records of adult patients admitted to any of five hospitals within the Johns Hopkins Hospital network (excluding labor and delivery admissions) from 2016 to 2021. Following the original AAM development paper^19^, the prediction outcome was a patient’s need for the ICU, defined as any in-hospital death, unexpected transfer to the ICU, or long stay in the ICU. The breakdown of the patient outcomes by hospital is shown in Supplementary Table 1. The AAM-inspired model uses predictors derived from laboratory tests, vital signs, patient demographics, and circumstances of admission (full list in the Supplementary Information). To train an encounter-level triage model, we used data from the first time point for each patient in order to discriminate between patients who deteriorate and patients who do not deteriorate. This differs from the original AAM model, which was trained to make hourly predictions for each patient.

We created training, validation, and test splits as follows: We first defined a multi-site evaluation test set by randomly selecting 20% of patient encounters from each hospital. This combined, multi-site evaluation dataset contained 60,998 patient encounters and was used in the application of the proposed evaluation framework. We defined per-site training datasets using the remaining 80% of patient encounters from each hospital. The AAM-inspired model was trained using only the Hospital 2 training dataset. This was intentionally done to mimic the common practical scenario in which a model is applied to data from new sites that were not used for the model’s development. The training datasets of the remaining hospitals were only used for model performance diagnostics in testing the effect of using more subgroup training data.

We applied Slice Finder^15^ using its publicly available implementation (https://github.com/yeounoh/slicefinder). We used a minimum slice sample size of 400 and a minimum effect size (Cohen’s *d* of 0.4).

#### Simulated data

We simulated data for a classification task using the following data generating process (DGP): We generated 10 iid discrete features *X*_1_, …, *X*_10_ ~ *Rademacher* in which the Rademacher distribution is the discrete distribution assigning probability 0.5 to the events *X*_*i*_ = 1 and *X*_*i*_ = −1. We then sampled the label according to $Y \sim Bernoulli\left(p=\sigma \left({X}^{T}\beta \right)\right)$, where $\sigma \left(z\right)=\frac{1}{1+{e}^{-z}}$ is the sigmoid function, and *β* are coefficients randomly sampled from the mixture of normal distributions $0.5{\mathcal{N}}\left(1,{0.5}^{2}\right)+0.5{\mathcal{N}}\left(-1,{0.5}^{2}\right)$. Note that the DGP is random, and was generated 50 times to create Fig. 6a.

This DGP generates data which can be accurately fit by a logistic regression model with coefficients *β*. Thus, we took a logistic regression model fit to 50,000 samples from the DGP as the target model to evaluate using the subgroup discovery algorithms. We generated an evaluation dataset using 10,000 samples from this DGP. To create a problematic subgroup in the evaluation dataset, without loss of generality, we selected the subgroup *X*_2_ = − 1 ∧ *X*_3_ = 1 (which consists of 25% of the data) and randomly flipped the labels of samples in this subgroup with probability 0.5. For the scalability experiment, we modified the DGP by generating various numbers of features {25, 50, 250, 500, 2500, 5000} and performing five trials for each.

## Supplementary information

Supplementary Information

## Data Availability

The data are not publicly available because they are from electronic health records approved for limited use by Johns Hopkins University investigators. Making the data publicly available without additional consent, ethical, or legal approval might compromise patients’ privacy and the original ethical approval. To perform additional analyses using this data, researchers should contact S.S. to apply for an IRB-approved research collaboration and obtain an appropriate data use agreement.

## Code availability

Code for implementing and demonstrating the AFISP workflow is available at https://github.com/asubbaswamy-fda/afisp. In this repository we provide a demo of the software on simple, publicly available data, as well as provide the code used in the experiments in the main paper.
