# Supplementary material for: A data-driven framework for identifying patient subgroups on which an AI/machine learning model may underperform
Source: NPJ Digit Med. 2024 Nov 21;7:334. doi: 10.1038/s41746-024-01275-6 (PMC11582698; doi:10.1038/s41746-024-01275-6)
Supplement: Supplementary file 1 — Supplementary Information [file 41746_2024_1275_MOESM1_ESM.pdf]

Supplementary Information

Supplementary 1. AAM-INSPIRED MODEL EXPERIMENT DETAILS

Supplementary 1A. Cohort characteristics

	Hospital 1	Hospital 2	Hospital 3	Hospital 4	Hospital 5	Total
**Patient Encounters**	43,456	44,545	117,210	52,927	46,839	304,977
**Outcome**	1,793 (4.1%)	878 (2.0%)	10,435 (8.9%)	1,662 (3.1%)	769 (1.6%)	15,537 (5.1%)
**Number of Beds**	420	225	1,091	230	245	—
**Trauma Level**	Level 2	N/A	Level 1	Level 2	N/A	—

Supplementary Table 1. Summary of outcomes of patient cohort by hospital, and hospital characteristics. Outcome refers to the patient outcome, "ICU need", which is the prediction target of the AAM model.

Supplementary 1B. AAM-Inspired Model features and description

In our custom implementation of the AAM model, inspired by the AAM model developed in [1], we used the subset of the original features available in the dataset. Table 2 lists the features we used in our custom implementation of the AAM model, as well as the feature transformations (again following the transformations used in [1]). The main differences from [1] are that we did not include composite indices (LAPS2 and COPS2 [2, 3], custom in-house scores created by the same organization that developed the AAM model), per-hospital indicators, and we trained the model to predict at the encounter level. We did not include the per-hospital indicators in our custom implementation of the AAM model because we wanted to apply it to data coming from hospitals that were not used in its development. Additionally, we trained the model to predict at the encounter level for simplicity of enabling encounter level analysis. For exact details regarding extraction of features used in the AAM model, please consult Section 2 and Table 1 of [1].

The AAM-inspired model is a logistic regression. We trained it using the `scikit-learn` Python package [4], with the inverse l2 regularization strength hyperparameter C set to 1000.

Supplementary 1C. Baseline model

The baseline model was selected to have overall performance comparable to, but worse than, the AAM-inspired model. To have some correspondence to a real-world model, we trained the baseline model using a subset of the AAM-inspired model's features that correspond to features used by the National Early Warning Score (NEWS) [5], a frequently used patient deterioration risk score. The 15 features used were systolic blood pressure instability, latest systolic blood pressure, latest heart rate, heart rate instability, oxygen saturation instability, latest oxygen saturation, worst oxygen saturation, respiratory rate instability, latest respiratory rate, worst respiratory rate, temperature instability, latest temperature, and latest Glasgow Coma Scale (GCS), age, and sex. The feature definitions and transformations were the same as those used by the AAM-inspired model in Table 2.

To achieve more competitive performance with the AAM-inspired model despite the reduced feature set, the baseline model was trained as a gradient boosted machine (GBM) using the `LGBM` package [6]. After using `FLAML` for automatic hyperparameter tuning, the selected hyperparameters were: number of estimators = 200, number of leaves = 139, minimum samples per child = 8, and learning rate = 0.05. The baseline model achieved an AUROC of 0.944 (CI 0.940, 0.950) on the full multi-site evaluation dataset.

Supplementary 1D. Subgroup definition features

The features listed in Table 3 were selected for the subgroup defining feature set in the Stability Analysis step of AFISP. Comorbidities were extracted from ICD-9 codes. In total there were 91 features.

**Feature**	**Transformation**
Laboratory Tests	
Anion gap	Linear
Bicarbonate	Quadratic
Glucose	Linear
Hematocrit	Cubic
Lactate	Linear
Log blood urea nitrogen	Linear
Log creatinine	Quadratic
Sodium	Linear
Troponin	Linear
Troponin missing flag	Indicator
Total white blood cell count	Linear
Vital Signs	
Latest diastolic blood pressure	Quadratic
Instability (i.e., highest - lowest in last 24 hours) of systolic blood pressure	Linear
Latest systolic blood pressure	Cubic
Latest heart rate	Cubic
Log heart rate instability	Quadratic
Log oxygen saturation instability	Linear
Logit ($log(\frac{x}{1-x})$ latest oxygen saturation	Cubic
Logit worst oxygen saturation	Linear
Log respiratory rate instability	Linear
Log temperature instability	Quadratic
Latest temperature	Quadratic
Latest respiratory rate	Cubic
Worst respiratory rate	Linear
Latest neurological status (Glasgow Coma Scale)	Linear
(Anion gap $\div$ serum bicarbonate) $\times$ 1000	Linear
Shock index (latest heart rate $\div$ latest systolic blood pressure)	Linear
Other	
Logit transpired length of stay	Linear
Logit age	Quadratic
Sex	Female indicator
Time of day	Time frame 1: 01:00-07:00<br>Time frame 2: 07:00-12:00<br>Time frame 3: all else
Admit category	1 ED, Surgical<br>2 Non-ED, Surgical<br>3 ED, Medical<br>4 Non-ED, Medical

Supplementary Table 2. Features used in the AAM-inspired model.

**Feature**
Abnormal lung finding
Acute bronchitis
Acute liver disease
Acute pancreatitis
Acute pulmonary heart disease
Acute respiratory failure
Admit source
Admitted from Emergency Department (ED)
Age
AIDS
Anemia
Acute respiratory distress syndrome (ARDS)
Asthma
Bladder cancer
Bronchiectasis
C. diff
Cardiac arrest
Cardiogenic shock
Cerebrovascular disease
Chest pain
Chronic airway obstruction
Chronic bronchitis
Chronic kidney disease
Chronic pancreatitis
Chronic pulmonary
Chronic pulmonary heart disease
Chronic respiratory failure
Congestive heart failure
Convulsions
Chronic obstructive pulmonary disease (COPD)
Cough
Dementia
Diabetes
Diabetes with complications
Diabetes without complications
Dialysis
Dyspnea

Supplementary Table 3 continued from previous page

**Feature**
Emphysema
Epilepsy
End-stage renal disease (ESRD)
Gastrointestinal (GI) bleed
Heart arrhythmias
Heart attack
Heart failure
Hematologic malignancies
Hemiparesis
Hemoptysis
Hospital size large (>500 beds)
Hospital trauma level
Hypersensitivity pneumonitis
Hypoxemia
Immunocompromised
Immunodeficiency
Infections angus
Kidney cancer
Liver disease
Malignancy
Metastatic carcinoma
Metastatic solid tumor
Mild liver disease
Myocardial infarction
Nonspecific lung disease
Obesity
Obstructive sleep apnea
Organ insufficiency
Other shock
Pancreatic cancer
Peptic ulcer disease
Peripheral vascular disease
Pneumonia
Post-surgery trauma respiratory failure
Prostate cancer
Pulmonary embolism

Supplementary Table 3 continued from previous page

Feature
Renal disease
Renal insufficiency
Respiratory prob external agents
Rheumatic disease
Season 1 (Nov, Dec, Jan, Feb)
Season 2 (Mar, Apr, May, Jun)
Season 3 (Jul, Aug, Sep, Oct)
Sepsis
Septic shock
Severe liver disease
Severe sepsis
Sex
Sickle cell crisis
Sickle cell trait
Sickle cell without crisis
Sleep apnea
Stroke
Transient ischemic attack

Supplementary Table 3. Features selected for creating possible subgroup definitions in the first step of AFISP.

Supplementary 2. AN ADDITIONAL EXPERIMENT ON THE MIMIC DATASET

To further test the applicability of AFISP, we applied AFISP to the publicly available MIMIC-III dataset [7]. For this experiment, we considered evaluating our own implementation of the SICULA model [8], a machine learning model intended to predict mortality in ICU patients based on their first 24 hours in the ICU.

Supplementary 2A. Features

This model uses the same types of features as the SAPS-II score [9]: the min and max vital sign values in the first 24 hours in the ICU for heart rate, systolic blood pressure, temperature, blood urea nitrogen, white blood cell count, potassium, sodium, bicarbonate, bilirubin, Glasgow coma scale, partial pressure of oxygen, and fraction inspired oxygen. Additionally, it uses patient age, the type of admission (scheduled surgical or emergency), and indicators for if the patient had HIV/AIDS, metastatic cancer, or a hematologic malignancy.

Supplementary 2B. Dataset

The extracted dataset consisted of 34,386 adult patient encounters in the ICU at the Beth Israel Deaconess Medical Center between 2001 and 2012. 80% of the data was used to train the SICULA model while 20% was heldout for the evaluation set (6,878 patients in the evaluation set). The prevalence of the outcome (mortality) was 0.096 in the entire cohort.

Supplementary 2C. Subgroup Definition Features

We selected the following features for the subgroup defining features used in the stability analysis step of AFISP: patient sex, ethnicity (Black, Asian, Hispanic, White, or Other), age, insurance

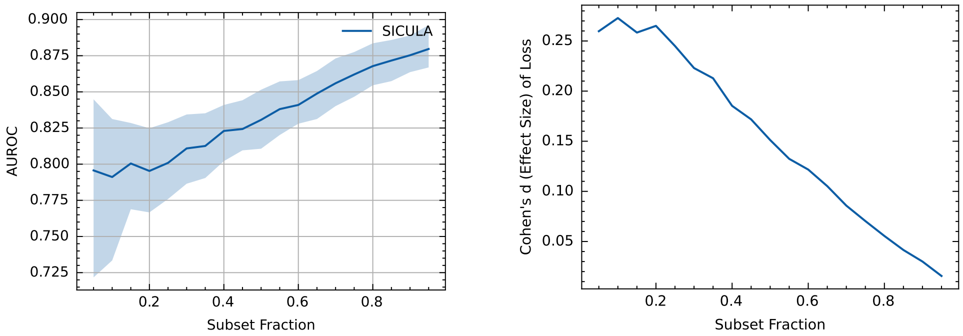

(a)

(b)

Supplementary Figure 1. (a) Performance stability curve of the SICULA model with respect to a shift in patient subgroup prevalence as measured by AUROC. Performance of the SICULA model decays from 0.880 (CI 0.867, 0.896) on the full evaluation set to 0.796 (CI 0.722, 0.845) on the worst 5% subset. The shaded region represents a 95% bootstrap confidence interval. (b) Plot of the effect size (Cohen's d) for the worst-case subsets of each subset fraction size. Because we did not have a reference performance threshold, we instead selected the worst-case subset based on the one with the highest effect size (subset fraction of 0.1).

(Medicaid, Medicare, Private, or Self Pay), first care unit (coronary care unit, medical ICU, surgical ICU, cardiac surgery care unit, and trauma surgical ICU), and admission type (emergency or not).

Supplementary 2D. Results

We analyzed how the performance of the SICULA model decays as the evaluation data distribution is gradually changed adversarially through shifts in the prevalence of subgroups defined with respect to the six features defined above. The resulting performance stability curve is shown in Figure 1a. As expected, performance of the SICULA model decays from an AUROC of 0.880 (CI 0.867, 0.896) on the full evaluation set to an AUROC of 0.796 (CI 0.722, 0.845) on the worst 5% subset.

In this experiment, we did not have a reference performance threshold to compare to (as opposed to the baseline model that was compared to in the AAM-inspired model experiment). Thus, we selected the worst-case subset based on the one with the highest effect size, which was the one with a subset fraction of 0.1 (Figure 1b).

We applied SIRUS to determine interpretable subgroup phenotypes. We allowed for up to three features to be simultaneously considered in a phenotype definition. After filtering subgroups based on significance (and correcting for multiple comparisons) and effect size, AFISP recovered the 6 subgroups reported in Table 4 (ordered by within-subgroup AUROC). The size of the subgroups are all quite large, ranging from 23% to 42% of the full evaluation set.

Supplementary 2E. Comparison to other algorithmic approaches

As in the AAM experiment, we also compared the subgroups found by AFISP to those found by SliceFinder (SF), a state-of-the-art algorithmic approach which searches all possible subgroups for those with poor performance. For this dataset we found that degree 3 slices (i.e., subgroups involving 3 features) were necessary to detect poor performance. Thus, we set (SF) to search the 225, 151 possible degree 3 slices. SF found 320 slices, which is much more than the 6 found by AFISP.

To enable the comparison, we jointly embedded the AFISP subgroups, SF slices, and 500 slices randomly sampled from the 225, 151 possible degree 3 slices into the same vector space using Partial Least Squares regression. The resulting loading plot is shown in Figure 2. The plot captures correlations between slices and performance: while the random slices (blue points) are distributed throughout the space, the SF slices are concentrated in the right two quadrants of the space.

The space also captures similarities between subgroups. For example, the vectors for AFISP subgroups 3, 5, and 6 are closely aligned, and these subgroups deal with the intersection of patients older than 66, whose first care unit was not the cardiac surgery intensive care unit, and whose admission was related to an emergency, unscheduled visit.

Subgroup #	Phenotype	AUROC [95% Bootstrap CI]	N
1	Age >= 75.8 & Medicare & not CSRU	0.81 [0.78, 0.83]	1570
2	Medicare & not CCU & not CSRU	0.81 [0.79, 0.83]	2208
3	Age>= 66.1 & not CSRU & Emergency Admission	0.82 [0.80, 0.84]	2439
4	Medicare & not CSRU & Emergency Admission	0.82 [0.80, 0.84]	2673
5	Age >= 66.1 & not CSRU	0.82 [0.88, 0.84]	2636
6	Age >= 66.1 & Emergency Admission	0.83 [0.81, 0.85]	2887

Supplementary Table 4. Subgroups found by AFISP on the MIMIC dataset

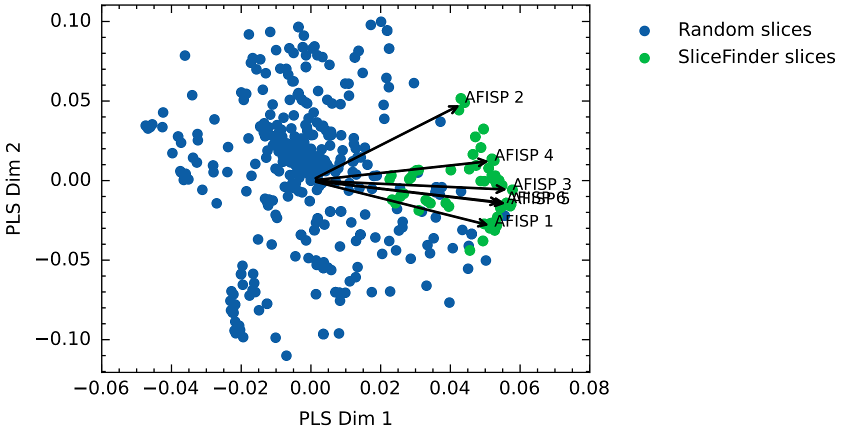

Supplementary Figure 2. Loading plot of the first two dimensions created by jointly embedding subgroups found by SliceFinder (SF) (green points), random candidate subgroups (blue points), and subgroups found by AFISP (portrayed as black vector direction arrows) using partial least squares (PLS) to predict model loss. While the random slices are spread throughout the space, all SliceFinder slices are in the right two quadrants, indicating that PLS was able capture subgroup-performance correlations. Further, the AFISP vectors "cover" the SF region of the space, capturing the relevant directions in only 6 subgroups as opposed to the 328 found by SF.

Finally, the 6 AFISP subgroup vectors do a good job of capturing the relevant directions in the space associated with the SF slices. Thus, AFISP found a concise set of subgroups that cover the slices selected by SF.

SUPPLEMENTARY REFERENCES

1. P. Kipnis, B. J. Turk, D. A. Wulf, J. C. LaGuardia, V. Liu, M. M. Churpek, S. Romero-Brufau, and G. J. Escobar, "Development and validation of an electronic medical record-based alert score for detection of inpatient deterioration outside the icu," J. Biomed. Informatics **64**,

10–19 (2016).
2. G. J. Escobar, J. D. Greene, P. Scheirer, M. N. Gardner, D. Draper, and P. Kipnis, "Risk-adjusting hospital inpatient mortality using automated inpatient, outpatient, and laboratory databases," Med. Care pp. 232–239 (2008).
3. C. van Walraven, G. J. Escobar, J. D. Greene, and A. J. Forster, "The kaiser permanente inpatient risk adjustment methodology was valid in an external patient population," J. Clin. Epidemiol. **63**, 798–803 (2010).
4. F. Pedregosa, G. Varoquaux, A. Gramfort, V. Michel, B. Thirion, O. Grisel, M. Blondel, P. Prettenhofer, R. Weiss, V. Dubourg *et al.*, "Scikit-learn: Machine learning in python," The J. Mach. Learn. Res. **12**, 2825–2830 (2011).
5. G. B. Smith, D. R. Prytherch, P. Meredith, P. E. Schmidt, and P. I. Featherstone, "The ability of the national early warning score (news) to discriminate patients at risk of early cardiac arrest, unanticipated intensive care unit admission, and death," Resuscitation **84**, 465–470 (2013).
6. G. Ke, Q. Meng, T. Finley, T. Wang, W. Chen, W. Ma, Q. Ye, and T.-Y. Liu, "Lightgbm: A highly efficient gradient boosting decision tree," Adv. Neural Inf. Process. Syst. **30** (2017).
7. A. E. Johnson, T. J. Pollard, L. Shen, L.-w. H. Lehman, M. Feng, M. Ghassemi, B. Moody, P. Szolovits, L. Anthony Celi, and R. G. Mark, "Mimic-iii, a freely accessible critical care database," Sci. data **3**, 1–9 (2016).
8. R. Pirracchio, M. L. Petersen, M. Carone, M. R. Rigon, S. Chevret, and M. J. van der Laan, "Mortality prediction in intensive care units with the super icu learner algorithm (sicula): a population-based study," The Lancet Respir. Medicine **3**, 42–52 (2015).
9. J.-R. Le Gall, S. Lemeshow, and F. Saulnier, "A new simplified acute physiology score (saps ii) based on a european/north american multicenter study," Jama **270**, 2957–2963 (1993).
